# Exploratory analysis of miRNAs-21, -26a, -34a, -181c, -181d, and -485-5p as potential biomarkers for tumor treating fields sensitivity in primary glioblastoma cell cultures

**DOI:** 10.1007/s10014-025-00527-x

**Published:** 2026-01-06

**Authors:** Sina Hemmer, Mohamed Henia, Walter Schulz-Schaeffer, Ralf Ketter, Benjamin Landau, Joachim Oertel, Steffi Urbschat

**Affiliations:** 1 Department of Neurosurgery, Saarland University Medical Center, Building 90.5, Kirrbergerstraße 100, 66421 Homburg, Germany; 2 Institute for Neuropathology, Saarland University Medical Center, Homburg, Germany; 3 Department of Neurology, Saarland University Medical Center, Homburg, Germany

**Keywords:** Glioblastoma, Tumor treating fields (TTFields), MicroRNA (miRNA), Personalized glioblastoma therapy

## Abstract

Tumor Treating Fields (TTFields) are approved for glioblastoma (GBM) treatment, but predictive biomarkers remain unclear. This study evaluates TTFields effectiveness in primary GBM cell cultures and explores miRNA biomarkers in tumor tissue, plasma, and primary cell cultures. TTFields were applied to 21 primary GBM cell cultures for 72 h. Cell viability was assessed pre- and post-treatment, with parallel evaluations in control cultures. Expression levels of miRNAs-21, -26a, -34a, -181c, -181d, and -485-5p were analyzed in tumor tissue, plasma, and untreated/treatment-exposed cultures. Correlation analyses examined TTFields response and miRNA expression. Response rates varied, with a mean cell viability reduction of 48.53%. Expression of miRNA-26a in tumor tissue (*p* = 0.041, r = 0.502) and miRNAs-21, -26a, and -181c in untreated control cultures (p < 0.05) correlated with increased TTFields effectiveness. Linear correlations were observed for miRNAs-26a and -181c in untreated control cultures ([95% CI: 0.001938–0.01725, *p* = 0.016; 95% CI: 0.0000003935–0.0001641, *p *= 0.049). Individual GBM cell cultures respond differently to TTFields. Overexpression of miRNA-26a in native tumor tissue and overexpression of miRNAs-21, -26a and -181c in untreated control cell cultures were positively correlated with increased effectiveness of TTFields treatment.

**Supplementary Information:**

The online version contains supplementary material available at 10.1007/s10014-025-00527-x.

## Introduction

The standard therapy regimen of newly diagnosed glioblastoma (GBM) consists of gross total resection, radiotherapy and systemic chemotherapy [1]. In recent years, Tumor Treating Fields (TTFields) have been approved for application during maintenance chemotherapy for GBM [2, 3]. TTFields induce antitumoral effects by interfering with formation of mitotic spindle microtubules, induction of autophagy and promotion of cell membrane permeability and DNA damage, as well as facilitation of an antitumoral immune response [4–7].

TTFields have shown effectiveness throughout all subgroups of the EF-14 trial [2, 8]. However, individual effectiveness of the treatment differed between individual patients. Identification of reliable biomarkers for assessing treatment response to TTFields remains challenging with only *PTEN, NF1, EGFR* and *PIK3CA* as potentially associated with TTFields-efectiveness [9, 10]. Predicting effectiveness of TTFields prior to their application could improve cost efficiency for health care systems and support the patients’ decision to undergo treatment despite potential physical or mental hardship that the treatment could entail.

MicroRNAs (miRNAs) have gained significant attention as biomarkers in a multitude of malignancies and predictors of treatment responses across various tumor types, including GBM [11, 12]. MicroRNAs are short non-coding RNAs that exert a regulatory role in post-transcriptional modification of gene expression [13]. Associations of expression of miRNA-181d and -26a with treatment response to alkylating chemotherapy, as well as correlating expression levels of miRNA-181d in tumor tissue and corresponding plasma samples could recently be demonstrated [14, 15]. Plasma levels of miRNAs-21 and -26 were suggested as circulating biomarkers for GBM [16, 17]. Both miRNAs are known regulators of PTEN expression, while miRNA-21 is also an inhibitor of autophagy [18–20]. MiRNAs-181c and -485-5p are assumed to suppress *MCAK* (mitotic centromere associated kinesin gene) expression, a regulator of mitosis, and thereby improve prognosis in breast cancer patients [21]. In GBM cells, cell cycle arrest and mitotic catastrophe of irradiated GBM cells are promoted by miRNA-34, a tumor suppressor and transcriptional target of p53 [11, 22, 23]. However, an interplay between the TTFields-mechanisms of mitotic spindle disruption, autophagy, cell cycle arrest and the expression of the abovementioned miRNAs has not been explored, yet.

Previous *in vitro* biomarker studies investigating TTFields in glioblastoma have mainly utilized patient-derived GBM cell lines, lacking data on primary glioblastoma cell cultures that are crucial for approximating the individual tumor behavior *in situ* [24–28].

This exploratory study aims to investigate the potential of miRNAs-21, -26a, -34, -181c, -181d and -485-5p as biomarkers for treatment response to TTFields by correlating treatment effects on primary GBM cell cultures with miRNA expression levels in these cells and in corresponding native tumor tissue, untreated primary GBM cell cultures, and plasma samples from 21 GBM patients.

## Methods

This study was designed as an exploratory single-center study, approved by the Ethics Committee of the Saarland Medical Association (votum number 73/20) and performed in accordance with the Declaration of Helsinki. Written informed consent was obtained from all participants.

### Study population

Patients with newly diagnosed histologically confirmed GBM WHO grade 4 over 18 years of age with no additional known cancerous disease or pregnancy that underwent tumor resection were eligible for study inclusion.

### Study samples

Glioblastoma tumor tissue was obtained during tumor resection. Blood samples were drawn from an arterial or intravenous line intraoperatively before opening of the dura. Reference blood samples from healthy donors were previously drawn and analyzed again in the present study [14]. Histopathological diagnosis, as well as O-6-methylguanine-DNA methyltransferase (MGMT) promoter- and isocitrate dehydrogenase 1 (IDH1) mutation status were compiled by the Institute of Neuropathology of the Saarland University Medical Center.

### Primary glioblastoma cell cultures

Cultivation of primary glioblastoma cell cultures was performed according to the standard protocol of our group [29, 30]. The tumor tissue was minced with forceps and scissors and suspended with Dulbecco’s Modified Eagle Medium (DMEM; GIBCO®, Life Technologies, Darmstadt, Germany) containing 10% fetal calf serum, 1% non-essential amino acids and 1% penicillin/streptomycin. The cell suspension was distributed to cell culture flasks and placed in a CO_2_ incubator at 37 °C with 5% CO_2_ for 24 h. A change of the cell culture medium was performed twice per week. After the formation of a cell monolayer was registered, a change of cell culture medium was carried out every two to three days and incubation was continued in a CO_2_ incubator at 37°C with 5% CO2. The cell cultures were split when a confluence cell monolayer in the flasks was reached. An early passage (1 or 2) was utilized for further experiments.

### Assessment of cell viability

When a confluent cell layer of 70–90% per flask was reached, cells were trypsinized and evaluated regarding cell count and cell viability utilizing the LUNA 2™ Automated Cell Counter (BioCat GmbH, Heidelberg, Germany) in conjunction with Trypan Blue staining. For every sample, 10 µl of cell suspension were examined.

### Application of TTFields in vitro

TTFields were applied to the primary glioblastoma cell cultures with the inovitro™ TTFields Lab Bench System (Novocure, Haifa, Israel) according to a modified protocol after Porat et al. [31]. In brief, 500 μl of the cell culture suspension containing 10.000–20.000 cells were distributed into 24 specifically designed ceramic petri dishes for the inovitro™ system each containing a 22 mm glass coverslip, before 500 µl of DMEM were added. The petri dishes were placed in a CO_2_ incubator at 37 °C with 5 % CO_2_ for 24 h, before the culture medium was removed and replaced with 2 ml of fresh DMEM. Sterilized parafilm was then used as coverage for all petri dishes. The ceramic dishes were placed onto a base plate connected to a TTFields generator to be treated with TTFields in a CO_2_ incubator with 5 % CO_2_. Due to heat development from TTFields, the CO_2_ incubator was refrigerated to 18 °C, with a stable temperature of 37 °C within the petri dishes. TTFields were applied with the optimal TTFields frequency of 200 kHz for GBM treatment and a low intensity of 2 V/cm continuously for 72 h. For every tumor, control cultures were prepared identically to TTFields-treated cultures, but were placed in a separate CO_2_ incubator at 37°C with 5 % CO_2_ for 72 h without TTFields-treatment. After 72 h, treated and control cell cultures were trypsinized and evaluated for cell viability as described before.

### Evaluation of TTFields treatment response

After 72 h of TTFields treatment, absolute cell count and cell viability were assessed again as described before. The relative individual treatment response was calculated based on the cell viability in the treated tumor cell cultures and the corresponding control cultures using the following formula:

$$Relative Reduction of Viability = \frac{{Viability Control Culture \left( \% \right){-} Viability TTFields Culture \left( \% \right)}}{Viability Control Culture \left( \% \right)} x 100$$

### MicroRNA analysis

MicroRNA (miRNA) expression levels of miRNAs-21, -26a, -34a, -181c, -181d and -485-5p were assessed as relative expression in corresponding native tumor tissue, TTFields-treated cell cultures, untreated control cell cultures and blood samples.

- *miRNA isolation*

Plasma miRNAs were isolated using the miRNeasy Serum Plasma Kit (Qiagen, Hilden, Germany) based on the manufacturer’s instructions, while isolation of miRNA from native tumor tissue and cell cultures was conducted with the miRNeasy Mini kit (Qiagen, Hilden, Germany). RNA quantification was performed with the NanoDrop spectrophometer (Thermo Fisher Scientific inc. Kandel, Germany). All RNA was diluted to a concentration of 20 ng/µl.

- *Quantitative reverse transcription polymerase chain reaction*

First, reverse transcription was conducted using the TaqMan™ MicroRNA Reverse Transcription Kit (Applied Biosystems Life Technologies, Darmstadt, Germany) along with MiRNA-specific Stem-Loop-Primers (TaqMan™ Small RNA Assay, Applied Biosystems Life Technologies, Darmstadt, Germany), adhering to the manufacturer’s guidelines. A master mix was prepared for each reaction, comprising 4.16 μl of PCR Water, 1.5 μl of RT-Buffer, 1 μl of Multiscribe™ Reverse Transcriptase, 0.19 μl of RNase Inhibitor, and 0.15 μl of dNTP Mix (All TaqMan™ MicroRNA Reverse Transcription Kit, Applied Biosystems™, Life Technologies, Darmstadt, Germany). For each reaction, 5 µl of diluted isolated RNA, along with 3 µl of the specific miRNA Stem-Loop-primer, were combined and centrifuged for 10 to 20 s. Subsequently, the samples underwent incubation in a thermal cycler (PTC-200, MJ Research, Hessisch Oldendorf, Germany) following this protocol: 30 min at 16 °C, 30 min at 42 °C, and 5 min at 85 °C. Post-incubation, the samples were stored at − 20 °C until further processing.

The quantitative PCR (qPCR) was executed using the Taqman™ Gene Expression Mastermix (Applied Biosystems™, Life Technologies, Darmstadt, Germany) and fluorescence labeled miRNA-specific Taqman™ miRNA assay primers were used according to the manufacturer’s instructions. Seven primer-specific master mixes were prepared (one for each miRNA-primer, one for RNU48), each consisting of 5 μl of Taqman ™ Gene Expression Mastermix, 0.5 μl of Taqman ™ miRNA primer, and 3.5 μl of PCR-grade water per sample. The qPCR was performed with the StepOne System (Applied Biosystems™, Life Technologies, Darmstadt, Germany). All reactions were executed in triplicate along with a negative control, using RNU48 as an internal reference as described before [14].

The expression of miRNA was normalized to the RNU48 expression in the corresponding tumor, plasma and cell culture sample, respectively. The quantification of miRNA expression was conducted by calculating the relative expression with the comparative 2^-(ΔCT)^ method as follows:

$$\begin{aligned} & Relative expression tumor tissue = 2^{{ - \left( {Ct \left( {miRNA tumor tissue} \right) {-} Ct \left( {RNU48 tumor tissue} \right)} \right)}} \\ & Relative expression plasma = 2^{{ - \left( {Ct \left( {miRNA plasma} \right) {-} Ct \left( {RNU48 plasma} \right)} \right)}} \\ & Relative expression TTFields cell culture = 2^{{ - \left( {Ct \left( {miRNA TTFields cell culture} \right) {-} Ct \left( {RNU48 TTFields cell culture} \right)} \right)}} \\ & Relative expression control cell culture = 2^{{ - \left( {Ct \left( {miRNA control cell culture} \right) {-} Ct \left( {RNU48 control cell culture} \right)} \right)}} \\ \end{aligned}$$

### Statistical analysis

Statistical analysis was carried out with GraphPad Prism 9.5 (GraphPad Software, Boston, Massachusetts, USA). Shapiro Wilk Normality test was used to evaluate Gaussian’ distribution. Kruskal-Wallis test was used to determine significance of differences of cell viability between cell cultures before and after TTFields treatment and control cultures, respectively. Correlation was evaluated using Spearman’s rank correlation coefficient and linear regression analysis. Mann Whitney U test was carried out to determine differences in miRNA expression levels of patient plasma samples and healthy donor plasma samples. A p-value < 0.05 was considered statistically significant.

## Results

### Study population and baseline tumor characteristics

Between 01/2022 and 08/2023, 55 patients met the inclusion criteria. After drop-outs due to withdrawal of consent to participate in the study, revision of histological diagnosis or poor growth-behavior of the cell cultures, 21 GBM patients were subject of this study. The male to female ratio was 1.33:1 with a mean patient age of 64.57 years (SD ± 12.52). IDH1 wild type was confirmed for all tumors. MGMT promoter methylation was present in 10/21 tumors (47.6 %). Baseline tumor and patient characteristics are given in Table 1.

**Table 1:** Overview of tumor and patient variables, viability of cell cultures before (“Baseline”) and after TTFields treatment for 72 h (“After TTFields”) in %, viability of control cell cultures after 72 h of incubation (“Control cultures”) in % and calculated relative reduction of viability (“Relative reduction of viability”) in %

Tumor	Sex	Age	MGMT	IDH	Viability (%)			Relative reduction of viability (%)
					Baseline	After TTFields	Control cultures	
T1	f	56	+	−	38.60	5.63	11.35	50.40
T2	m	82	−	−	93.50	35.80	58.20	38.49
T3	m	42	+	−	76.00	29.60	77.60	61.86
T4	f	66	−	−	72.70	52.88	68.30	22.58
T5	f	71	+	−	87.10	48.05	78.40	38.71
T6	f	78	+	−	60.20	27.14	71.83	62.22
T7	m	69	−	−	85.50	52.70	86.60	39.15
T8	m	55	+	−	86.50	62.60	63.00	0.63
T9	m	83	+	−	92.00	33.50	60.00	44.17
T10	m	67	−	−	73.00	31.10	55.40	43.86
T11	f	82	−	−	79.00	7.88	24.21	67.45
T12	m	47	−	−	75.00	22.00	53.00	58.49
T13	m	75	+	−	75.00	16.47	46.58	64.64
T14	m	48	−	−	78.30	41.00	64.00	35.94
T15	f	74	+	−	53.00	40.00	56.00	28.57
T16	f	53	−	−	84.60	24.54	65.72	62.66
T17	m	53	−	−	65.00	13.00	50.00	74.00
T18	m	72	−	−	77.50	34.33	62.00	44.63
T19	f	58	−	−	90.00	15.00	69.00	78.26
T20	m	74	+	−	80.00	31.00	61.00	49.18
T21	f	51	+	−	75.00	28.00	60.00	53.33

*T1–T21* Tumors 1–21, sex and age of the corresponding patient. *MGMT* MGMT promoter methylation status: “+” = MGMT promoter methylated, “−“ = MGMT promoter unmethylated. *IDH* IDH mutation status: “+” = IDH mutation present, “−“ = no IDH mutation present

### Primary tumor cell cultures

A sufficient growth behavior was recorded in 21 primary glioblastoma cell cultures, which were subject of this study. Mean growing time for the first passage (P0) was 17.95 days (7–38 days). After the first splitting process (passage P1), 1/21 cell cultures was directly treated with TTFields, while 20/21 cell cultures were subject of treatment after the second splitting process (passage P2).

### TTFields application to primary cell cultures

Individual baseline values of cell viability and viability values after TTFields treatment and after the control period of 72h for control cell cultures, as well as individual values of relative reduction of cell viability after TTFields treatment are given in Table 1.

A mean viability before treatment of 76.07 % (SD ± 12.93 %) was recorded, while after 72 h of TTFields application it was reduced to 31.06 % (SD ± 14.76 %). In the control cultures, a mean viability of 59.15 % (SD ± 16.49 %) was recorded after 72 h of incubation.

**Fig. 1:**
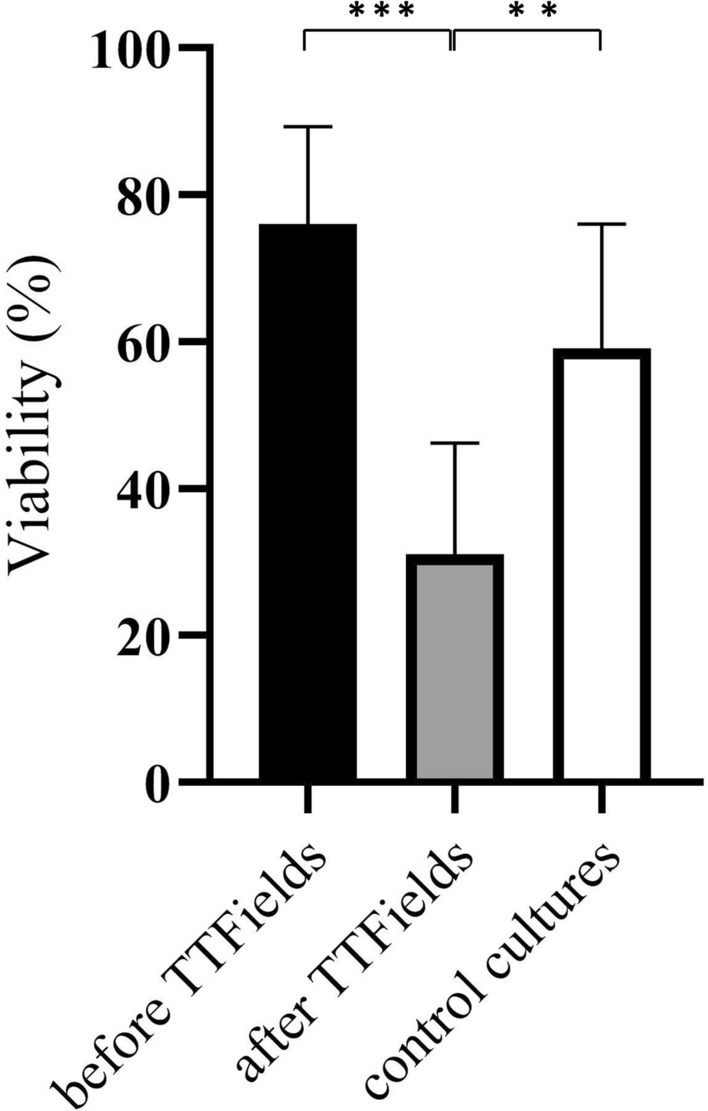
Comparison of cell viability of primary GBM cell cultures before and after TTFields treatment for 72 h and of control cell cultures at 72 h. Kruskal-Wallis test demonstrated a significant difference (*p* < 0.0001) between primary GBM cell cultures before and after TTFields treatment and between TTFields treated cell cultures and control cell cultures at 72 h. y axis: mean cell viability in %. x axis: experimental groups: “before TTFields” = primary GBM cell cultures before treatment with TTFields *in vitro*; “after TTFields for 72 h” = primary GBM cell cultures after 72 h of treatment with TTFields *in vitro*; “control cultures after 72 h” = corresponding primary GBM control cell cultures after 72 h. Whiskers indicate standard deviation. Asterisks indicate significance level

Calculation of the relative reduction of cell viability in TTFields-treated cultures versus control cultures confirmed differences in individual treatment response to TTFields, with a mean relative reduction of cell viability 48.53 % (SD ± 17.87 %), a minimum reduction of 0.63 % and a maximum reduction of 78.26 %. Kruskal-Wallis test confirmed a significant difference in cell viability before and after TTFields treatment (*p* < 0.0001) and between TTFields-treated cell cultures and control cell cultures (*p* = 0.0014), respectively (Fig. 1).

### MicroRNA analysis

- *microRNA expression levels*

The expression levels of miRNAs-21, -26a, -34a, -181c, -181d and -485-5p were measured in corresponding plasma, native tumor tissue as well as in TTFields-treated and untreated control cell culture samples (after 72 h of TTFields-treatment and incubation without treatment, respectively) of 21 GBM patients, and in plasma samples of 10 healthy blood donors. The median expression levels of miRNAs and range of values are given in Table 2.

**Table 2:** Overview of miRNA expression level (relative expression) median, minimum and maximum of miRNAs-21, -26a, -24a, -181c, -181d and -485-5p depending on the tissue of origin (plasma = patient plasma, TTFields = TTFields treated primary GBM cell cultures, native tumor = native tumor tissue, control cultures = primary GBM control cell cultures)

miRNA	Origin	n	Median	SD	Minimum	Maximum
21	Plasma	21	14.856030	323.937540	0.263405	1538.337453
21	TTFields	21	3.924571	4.505128	0.017450	15.399309
21	Native tumor	21	3.978366	10.982669	0.374220	49.684425
21	Control cultures	21	6.432481	191.136352	0.249028	908.905641
21	Healthy	10	2.752506	2.434938	2.163464	9.099283
26a	Plasma	21	8.560077	19.127730	0.427785	76.111067
26a	TTFields	17	0.044493	0.118662	0.000020	0.377633
26a	Native tumor	17	1.221079	0.878729	0.061041	2.705276
26a	Control cultures	21	0.212203	0.332552	0.000004	1.465382
26a	Healthy	10	1.644907	5.126149	0.221007	17.282870
34a	Plasma	21	0.431350	0.599843	0.064828	2.046271
34a	TTFields	20	0.250115	4.172050	0.000009	19.369245
34a	Native tumor	21	0.204356	0.377012	0.002028	1.614260
34a	Control cultures	13	0.278550	0.801836	0.046184	3.212637
34a	Healthy	10	0.365298	0.656070	0.014193	2.087410
181c	Plasma	17	0.079523	1.162076	0.004332	2.547990
181c	TTFields	20	0.001003	0.002774	0.000007	0.008912
181c	Native tumor	21	0.007576	0.008290	0.000260	0.032971
181c	Control cultures	21	0.001342	0.003382	0.000014	0.016465
181c	Healthy	10	1.345127	1.756937	0.149377	5.544070
181d	Plasma	21	0.258369	1.518594	0.010342	6.261007
181d	TTFields	20	0.090599	2.399843	0.005345	11.138034
181d	Native tumor	18	0.208084	0.269142	0.000002	1.029196
181d	Control cultures	21	0.071179	0.151329	0.016285	0.602039
181d	Healthy	10	0.594290	0.381867	0.076081	1.410474
485-5p	Plasma	17	0.145419	0.291758	0.001410	1.024225
485-5p	TTFields	20	0.003419	0.024617	0.000092	0.104929
485-5p	Native tumor	21	0.001446	0.001653	0.000001	0.007162
485-5p	Control cultures	20	0.003284	0.002798	0.000017	0.010510
485-5p	Healthy	9	0.071793	0.654788	0.004865	1.941976

*n* Number of samples, *SD* Standard deviation

**Fig. 2:**
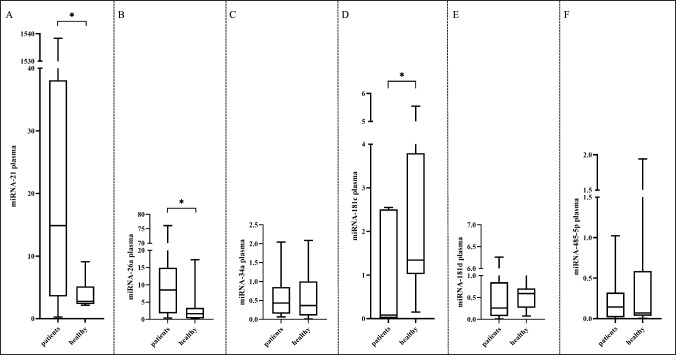
Results of Mann Whitney U test, comparison of plasma miRNA expression levels of **A** miRNA-21, **B** miRNA-26a, **C** miRNA-34a, **D** miRNA-181c, **E** miRNA-181d and **F** miRNA-485-5p between GBM patients and healthy individuals. y axis: range of relative expression of miRNA. Y-axis scales were individually adjusted for each miRNA to account for differences in expression ranges, allowing clearer visualization of expression relationships; x axis: patients = GBM patients, controls = healthy individuals. Boxes indicate interquartile range. Lines within boxes represent median value. Whiskers extend to the minimum and maximum values within 1.5 times the interquartile range from the lower and upper quartiles, respectively. Asterisk indicates significance (*p* < 0.05)

After exclusion of a Gaussian distribution through the Shapiro Wilk normality test, Mann Whitney U test demonstrated a significant distinction in plasma expression levels of miRNAs-21 (p = 0.042, U = 54), -26a (p = 0.021, U = 51) and -181c (*p* = 0.026, U = 41) between glioblastoma patients and healthy individuals (Fig. 2). Notably, expression levels for miRNA-181c were significantly higher in healthy individuals, compared to GBM patients, while expression levels of miRNA-21 and -26a were significantly higher in GBM patients.

### Correlation analysis

- *Correlation between miRNA expression levels from plasma, native tumor tissue and cell cultures*

Using Shapiro Wilk normality test, a Gaussian’ distribution for miRNA expression levels could not be demonstrated. A correlation matrix was generated to investigate the Spearman correlation coefficients between the miRNA expression levels of miRNAs-21, 26a, 34a, 181c, 181d and 485-5p in corresponding plasma, native tumor tissue and cell cultures samples (TTFields-treated cell cultures and control cell cultures after 72 h of incubation).

A positive correlation was recorded between expression levels of miRNA-26a (r = 0.62, *p* = 0.008) and miRNA-181d (r = 0.47, *p* = 0.045) in native tumor tissue and untreated control cell cultures, respectively. An additional positive correlation was revealed between expression levels of miRNA-34a in TTFields treated cell cultures and control cell cultures (r = 0.613, *p* = 0.038). No correlation was demonstrated between miRNA expression levels in plasma and tumor tissue, plasma and TTFields treated cell cultures, plasma and control cell cultures and native tumor tissue and TTFields treated cell cultures, respectively.

- *Correlation between miRNA expression levels and TTFields treatment response*

A correlation analysis of the different miRNA expression levels in plasma, tumor tissue and cell cultures (treated and untreated cultures) and the relative reduction of cell viability in the corresponding TTFields treated cell cultures was obtained using Spearman rank correlation analysis. MicroRNA expression levels in the untreated cell cultures were obtained after 72 h of incubation and served as a baseline reference for the untreated primary GBM cell cultures.

A significant positive correlation between expression levels of miRNA-26a in native tumor tissue (*p* = 0.041, r = 0.502), as well as expression levels of miRNA-21, 26a and 181c) in control cell cultures (*p* = 0.029, r = 0.474; *p* = 0.015, r = 0.522; *p* = 0.008, r = 0.561, respectively and the corresponding relative reduction in cell viability in TTFields treated cell cultures was revealed (Table 3). No correlation was observed between expression of the evaluated miRNAs and cell culture viability of untreated control cell cultures after 72 h of incubation.

**Table 3:** Results of Spearman correlation analysis between relative reduction of cell viability in TTFields treated cell cultures and miRNA expression levels of miRNAs-21, -26a, -34a, -181c, -181d and -485-5p in corresponding patient plasma, TTFields treated cell cultures, native tumor tissue and control cell cultures, respectively

Relative reduction vs miRNA	xy-pairs	Spearman r	95 % CI	*p* value
miRNA 21 plasma	21	− 0.3948	− 0.7129 to 0.05808	0.0765
miRNA 26a plasma	21	− 0.1156	− 0.5311 to 0.3448	0.6178
miRNA 34a plasma	21	− 0.05002	− 0.4821 to 0.4016	0.8295
miRNA 181c plasma	17	− 0.1057	− 0.5685 to 0.4080	0.6865
miRNA 181d plasma	21	− 0.139	− 0.5480 to 0.3237	0.548
miRNA 485-5p plasma	17	− 0.08088	− 0.5514 to 0.4287	0.7584
miRNA 21 TTFields	21	0.2416	− 0.2253 to 0.6182	0.2915
miRNA 26a TTFields	17	0.3162	− 0.2088 to 0.6997	0.2159
miRNA 34a TTFields	20	− 0.08571	− 0.5193 to 0.3829	0.7194
miRNA 181c TTFields	20	0.1188	− 0.3540 to 0.5433	0.6179
miRNA 181d TTFields	20	0.08872	− 0.3803 to 0.5215	0.7099
miRNA 485-5p TTFields	20	− 0.06316	− 0.5025 to 0.4021	0.7914
miRNA 21 tumor	21	0.1312	− 0.3308 to 0.5424	0.5709
miRNA 26a tumor	17	0.5025	0.01327 to 0.7976	0.0419
miRNA 34a tumor	21	0.3117	− 0.1520 to 0.6629	0.169
miRNA 181c tumor	21	0.2948	− 0.1701 to 0.6524	0.1945
miRNA 181d tumor	18	0.3767	− 0.1242 to 0.7246	0.1234
miRNA 485-5p tumor	21	0.2532	− 0.2134 to 0.6258	0.268
miRNA 21 control cultures	21	0.4742	0.03980 to 0.7578	0.0299
miRNA 26a control cultures	21	0.5222	0.1034 to 0.7838	0.0152
miRNA 34a control cultures	13	0.3329	− 0.2840 to 0.7549	0.2648
miRNA 181c control cultures	21	0.5612	0.1577 to 0.8041	0.0081
miRNA 181d control cultures	21	0.3248	− 0.1378 to 0.6710	0.1509
miRNA 485-5p control cultures	21	− 0.02338	− 0.4613 to 0.4237	0.9199

Column 1: miRNA and tissue of origin. xy-paris: data points for analysis, *CI* Confidence interval. *p* values < 0.05 indicated significance

**Fig. 3:**
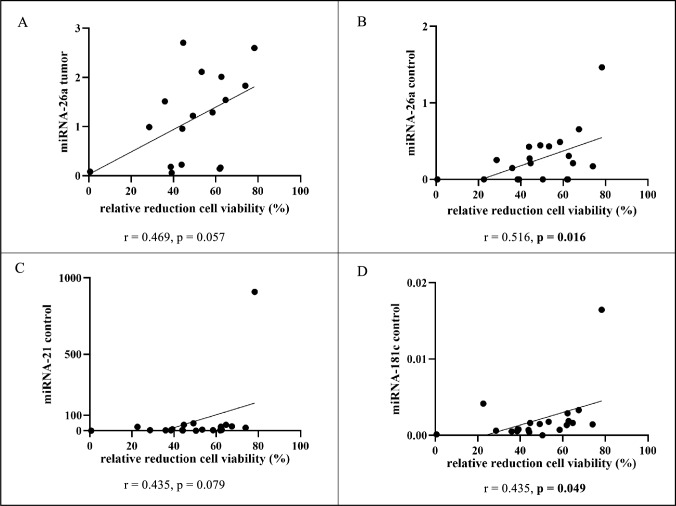
Results of linear regression analysis for **A** miRNA-26a in native tumor tissue and **B** miRNAs-26a, **C** -21, and **D** -181c in control cell cultures and relative reduction of cell viability in TTFields treated primary GBM cell cultures. y axis: relative expression of miRNA and tissue of origin (tumor = native tumor tissue, control cultures = control cell cultures); x axis: relative reduction of cell viability in % of TTFields treated primary GBM cell culture. r = correlation coefficient, *p* = level of significance

Linear regression analysis was conducted to evaluate a linear correlation between the relative reduction in cell viability and the expression levels of miRNA26a in tumor tissue and miRNAs 21, 26a and 181c in control cell cultures. Here, a linear correlation was demonstrated between relative reduction in cell viability and miRNAs-26a (r = 0.516 [95% CI: 0.001938 to 0.01725], *p* = 0.016) and -181c (r = 0.435 [95% CI: 0.0000003935–0.0001641], *p* = 0.049) in control cell cultures. No linear correlation was found between relative reduction in cell viability and expression of miRNA-26a in native tumor tissue (r = 0.469 [95% CI: − 0.00085 to 0.046], *p* = 0.057) and miRNA-21 in control cultures (r = 0.435 [95% CI: − 0.539 to 8.912], *p* = 0.079), respectively (Fig. 3).

A notable overexpression of miRNAs-21, -26a and -181c was recorded for tumor T19 with the most significant concurrent relative reduction of cell viability (78.26 %) amongst all tumors in this study.

We performed a supplementary analysis to examine whether changes in miRNA expression after TTFields treatment were associated with treatment response. For each tumor culture and for each miRNA separately (miR-21, miR-26a, miR-34a, miR-181c, miR-181d, and miR-485-5p), we calculated the absolute reduction in expression after 72 h of TTFields exposure compared with matched control cultures (ΔmiRNA = miRNA level in control cultures − miRNA level in TTFields-treated cultures). We then correlated this ΔmiRNA value with the relative reduction in cell viability observed in the corresponding TTFields-treated cultures.

Across all 21 primary tumor cultures, a significant association was identified for miR-26a, where larger reductions in miR-26a expression after TTFields exposure correlated with greater decreases in cell viability (Spearman r = 0.49, *p* = 0.046; linear regression analysis r = 0.57 [95% CI: 0.13 to 0.83], *p* = 0.016). For the remaining miRNAs (miR-21, miR-34a, miR-181c, miR-181d, and miR-485-5p), no significant correlations were observed. (Supplementary Fig. 1)

## Discussion

This study highlights differences in the individual response rates of primary GBM cell cultures to TTFields treatment ranging between 0.63 % and 78.26 %, with a mean relative reduction of cell viability of 48.53 %. miRNA-21, -26a, and -181c expression levels in plasma from GBM patients differed significantly from healthy individuals. Positive correlations were revealed between miRNA-26a and -181c in tumor tissue and corresponding primary cell cultures. Overexpression of miRNA-26a in native tumor tissue, as well as overexpression of miRNAs-21, -26a and -181c in untreated control cell cultures were positively correlated with an increased relative reduction of cell viability and thus increased sensitivity of primary GBM cell cultures to TTFields.

### Primary GBM cell cultures demonstrate individual sensitivity to TTFields

This study presents novel data on the specific impact of TTFields on 21 patient-derived primary GBM cell cultures in their initial or secondary passages, marking a departure from prior investigations focused on cell lines [24, 27, 28, 32].

Clark et al. previously attempted to explore TTFields in *in vitro* models using patient-derived cell cultures, yet their use of passages 5 to 40 did not adequately preserve the essential characteristics of primary cultures in earlier passages [24]. Nickl et al. demonstrated the efficacy of TTFields in three primary GBM cell cultures, three 3D GBM organoid models and two cultivated tumor slices form eight different patients, with a particular emphasis on the expression of Ki67 and the corresponding treatment response. Their investigation revealed significant variations in the treatment response to TTFields and Ki67 expression, serving as a proof-of-principle on a smaller cohort [28].

In accordance to the *in vitro* data from Nickl et al. and the clinical data from the EF-14 trial that suggest an overall benefit of the therapy but with highly individual effectiveness of TTFields, our data confirms differences in the individual sensitivity of primary glioblastoma (GBM) cell cultures to TTFields treatment ranging between 0.63 % and 78.26 %, with a mean relative reduction of cell viability of 48.53% [2, 28]. 

### Correlation of miRNA-expression levels between plasma and tumor cells

This study found significantly different plasma levels of miRNAs-21, -26a, and -181c in GBM patients versus healthy controls. Overexpression of miRNAs-21 and -26a in GBM patients was revealed, while miRNA-181c was underexpressed. No correlation was found between plasma and tumor tissue, likely due to GBM heterogeneity and miRNA susceptibility to confounders [33–35]. Notably, while miRNA-181c overexpression in GBM control cultures increased TTFields sensitivity, higher plasma levels were observed in healthy individuals. This previously unreported phenomenon lacks a clear underlying mechanism and requires further investigation.

### MicroRNAs as biomarkers for effectiveness of TTFields in vitro

- *microRNA-21 and microRNA-26a*

Current evidence for TTFields response biomarkers is limited, with *PTEN* (phosphatase and tensin homolog gene) mutation being the only suggested marker in recurrent GBM. *PTEN*, a glioma tumor suppressor gene, regulates spindle architecture and chromosome alignment, but mutations in ~50% of GBM cases shorten survival [36–38]. Its wild-type form inhibits the PI3K/AKT pathway and helps regulate cell proliferation and survival. Loss or mutation of *PTEN* is associated with tumor progression and treatment resistance, but it may also affect how tumor cells respond to certain therapies [18, 20, 39]. In our study, we observed increased sensitivity to TTFields in cell cultures with elevated miRNA-21 and miRNA-26a expression, which seems counterintuitive since both of these miRNAs have been linked to reduced *PTEN* expression [18, 20]. Interestingly, Dono et al. found that patients with PTEN-mutant recurrent glioblastomas appeared to benefit more from TTFields than those with wild-type *PTEN* [10]. One possible explanation, although still speculative, is that loss of *PTEN*, whether through mutation or miRNA regulation, might disrupt mitotic processes and make tumor cells more vulnerable to the effects of TTFields. This could help explain our findings, but more experimental work at the cellular and molecular level will be needed to explore this further.

MiRNA-21 also inhibits autophagy, a key mechanism in TTFields-induced cell death [6, 19, 40]. While TTFields trigger autophagy as a survival response, autophagy inhibition sensitizes glioblastoma cells to therapy, aligning with our findings. Further research is needed to clarify the role of *PTEN*-related miRNA regulation in TTFields response.

In addition, our supplementary analysis showed that the magnitude of miRNA modulation after TTFields exposure may itself be biologically meaningful. Specifically, we found that a greater reduction in miR-26a expression after 72 h of TTFields treatment (ΔmiR-26a) was significantly correlated with a larger decrease in cell viability across 21 primary tumor cultures. Notably, this association was not observed for the other miRNAs assessed (miR-21, miR-34a, miR-181c, miR-181d, and miR-485-5p), suggesting a potential miR-26a–specific link to TTFields sensitivity. This finding may reflect selective elimination of miR-26a-high cellular subpopulations or a functional contribution of miR-26a downregulation to treatment response, both of which are hypotheses that merit further mechanistic investigation.

- *microRNA-34a*

Cell cycle arrest and mitotic spindle disruption represent key players in TTFields mechanisms. While miRNA-34 promoted these mechanisms in irradiated GBM cells in earlier studies, expression levels of miRNA-34a did not correlate with relative reduction of cell viability and thus effectiveness of TTFields in our study [11, 22, 23]. Correlating levels of miRNA-34a expression in TTFields treated cell cultures and untreated control cultures could even indicate that miRNA-34a is not involved in the regulation of TTFields-induced mechanisms.

- *microRNA-181c and microRNA-485-5p*

MiRNA-181c and -485-5p are suggested to suppress expression of *MCAK* (mitotic centromere-associated kinesin), a key regulator of microtubule dynamics and chromosome segregation during mitosis [21]. In breast cancer, overexpression of these miRNAs has been associated with reduced MCAK levels and improved patient survival [41]. In our study, miRNA-485-5p expression did not show a correlation with TTFields sensitivity. However, we observed a positive linear correlation between miRNA-181c levels in GBM control cell cultures and increased sensitivity to TTFields. While these findings hint at a possible synergy between miRNA-181c-mediated MCAK regulation and TTFields’ disruption of mitotic processes, these assumptions remain highly speculative and require further validation through targeted experimental studies. Considering the exploratory nature of our work, we present these observations as preliminary hypotheses that could inform future investigations.

- *microRNA-181d*

Former studies introduced miRNA-181d as a prognostic biomarker in GBM patients receiving alkylating chemotherapy with correlating expression levels in plasma and tumor tissue of GBM patients [42, 43]. These results could not be reproduced by the study at hand and miRNA-181d is not suggested to be a biomarker for effectiveness of TTFields in GBM cells.

### Limitations

The study’s statistical analysis is limited by the small sample size. While correlations were found between relative reduction of cell viability after TTFields treatment and miRNA-26a in tumor tissue, as well as miRNAs-21, -26a, and -181c in untreated GBM cultures, outliers may skew results. Baseline miRNA expression was not assessed before TTFields treatment but from untreated control cultures after 72 h. These controls are assumed to accurately reflect tumor biology and were used as baseline references. The lack of correlation between miRNA expression and control culture viability after 72 h suggests that miRNA expression did not independently influence viability decline outside of TTFields treatment.

Given the exploratory nature of this study with a focus on primary cell cultures, no experiments with knockdown or overexpression of the miRNAS in established glioblastoma cell cultures were conducted. In order to corroborate the findings of this study, these experiments would be necessary to establish basic causality.

## Conclusion

This study is the first to report individual effects of TTFields on a larger number of primary GBM cell cultures. In this exploratory analysis, overexpression of miRNA-26a in native tumor tissue, as well as overexpression of miRNAs-21, -26a and -181c in primary GBM cell cultures correlated with an increased sensitivity of GBM cells to TTFields *in vitro*, indicating their potential as biomarkers to predict a treatment response to TTFields. Clinical studies are required to explore if the effectiveness of TTFields can be predicted by the treatment response of primary GBM cell cultures and to corroborate epigenetic findings with the clinical course of GBM patients treated with TTFields.

## Supplementary Information

Below is the link to the electronic supplementary material.

Scatter plots showing the relationship between relative reduction in cell viability (x-axis) and ΔmiRNA expression (y-axis) across 21 primary glioblastoma cultures. Separate panels are shown for miR-21 (A), miR-26a (B), miR-34a (C), miR-181c (D), miR-181d (E), and miR-485-5p (F). A linear regression line with 95% confidence band is displayed onlyfor miR-26a, as this was the only miRNA exhibiting a significant association in the statistical analyses. Supplementary file1 (PDF 283 kb)

## Data Availability

The data that support the findings of this study are not publicly available due to ethical and legal restrictions related to patient privacy. Data may be available from the corresponding author upon reasonable request and with appropriate approvals.
