# Supplementary figures and images for: Exploratory analysis of miRNAs-21, -26a, -34a, -181c, -181d, and -485-5p as potential biomarkers for tumor treating fields sensitivity in primary glioblastoma cell cultures
Source: Brain Tumor Pathol. 2026 Jan 6;43(3):85–96. doi: 10.1007/s10014-025-00527-x (PMC13375958; doi:10.1007/s10014-025-00527-x)

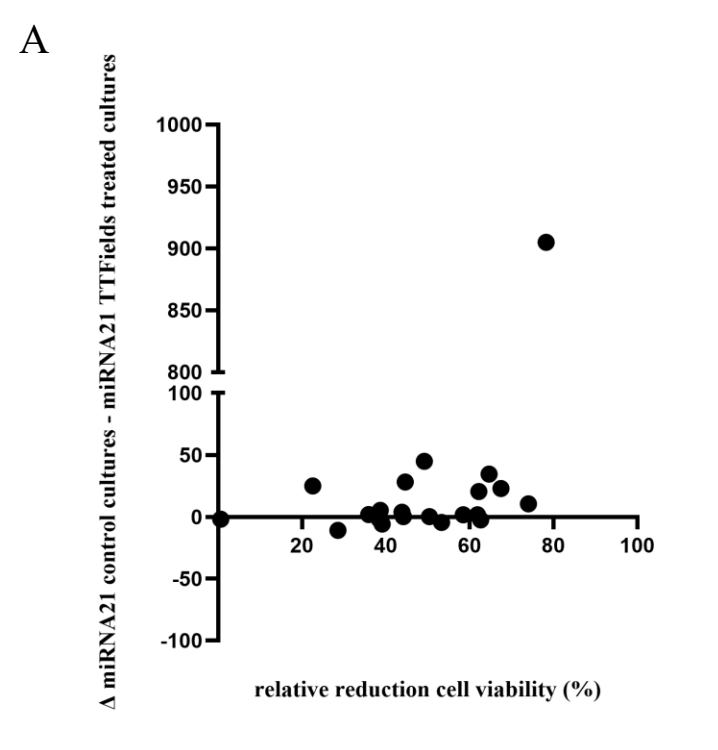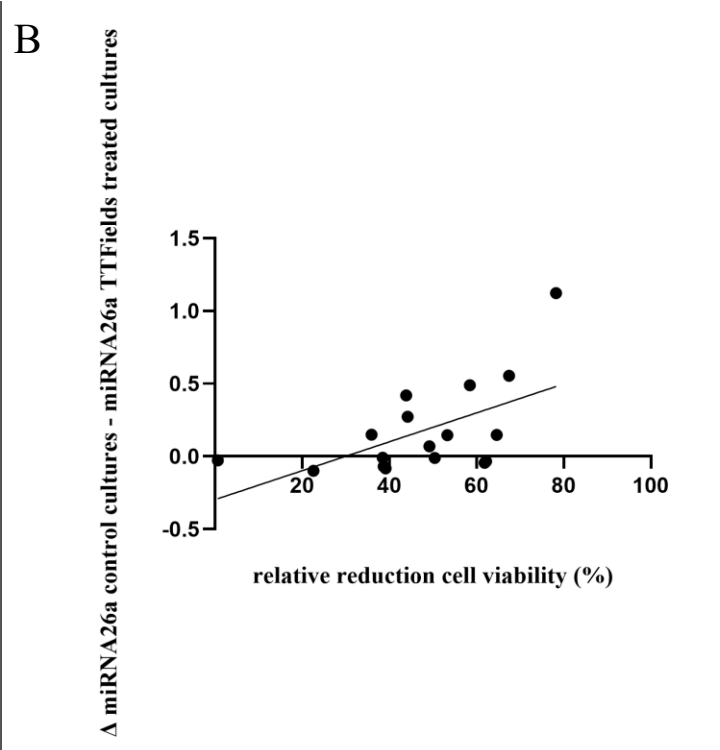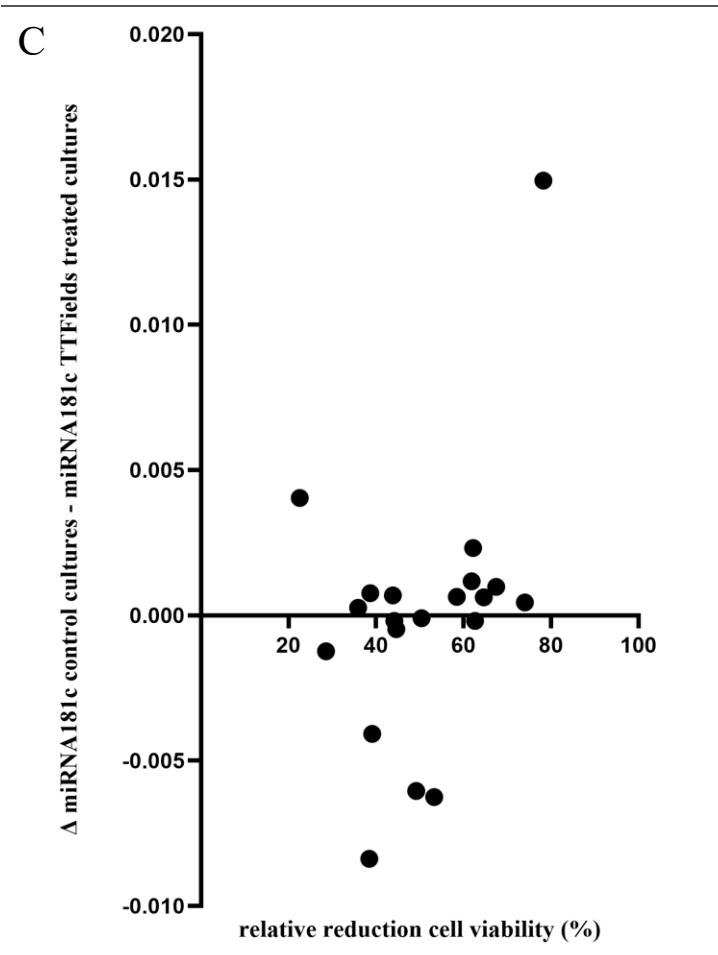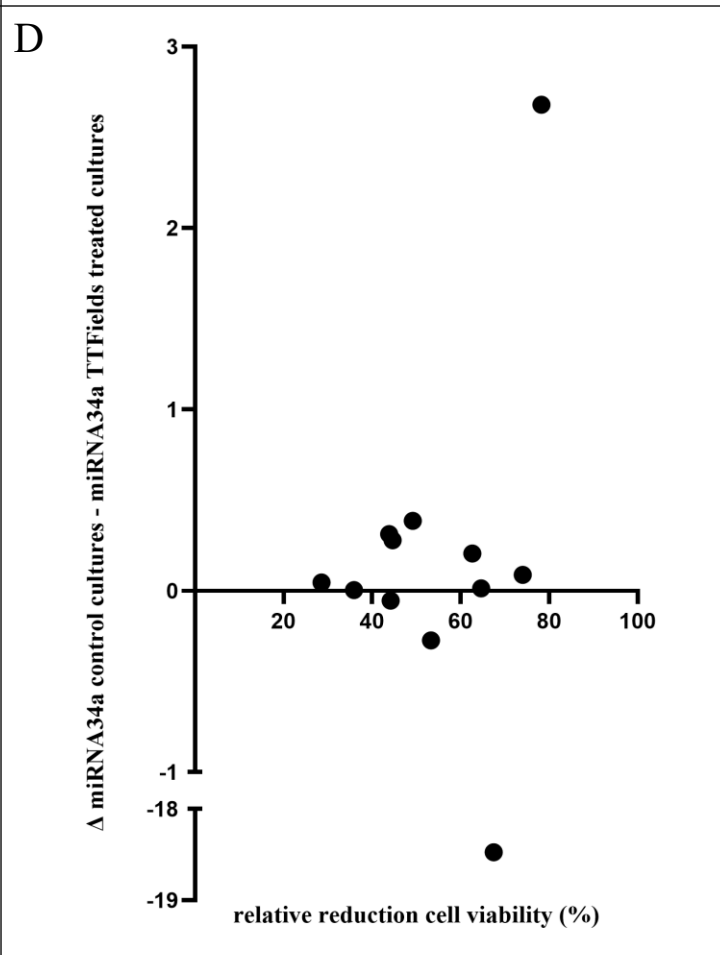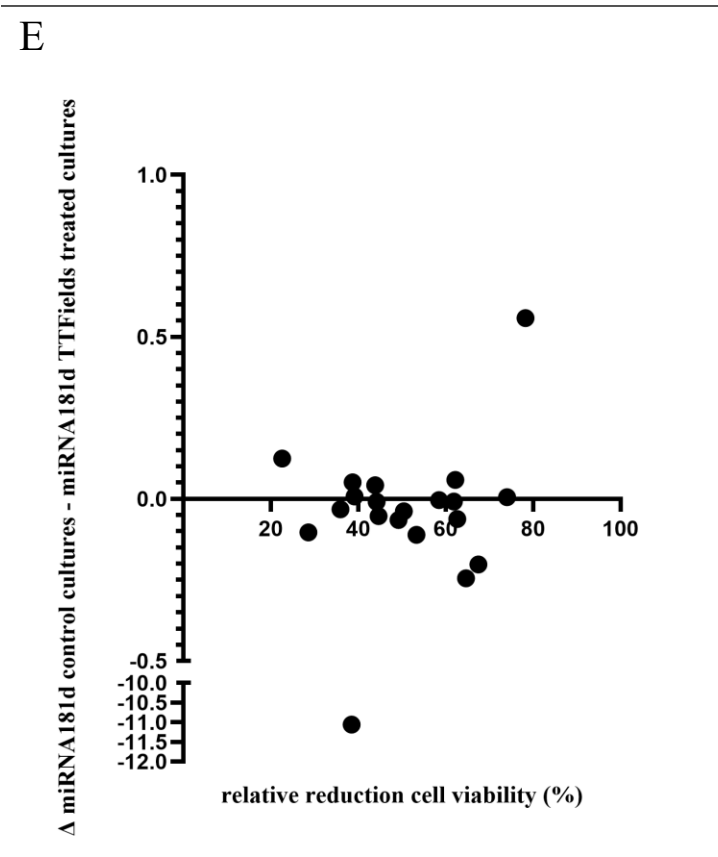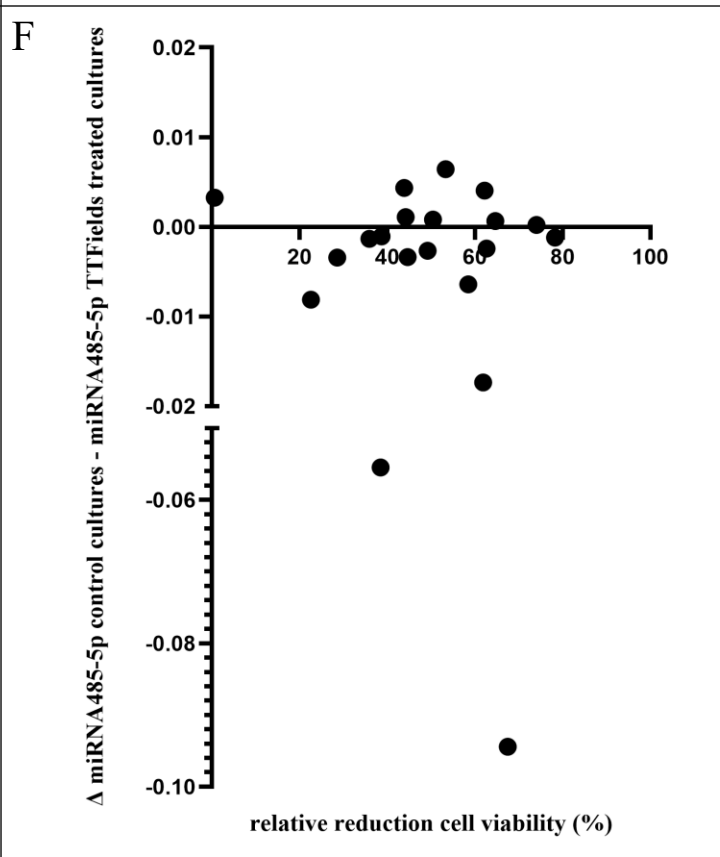

Supplement: Supplementary file 1 — Scatter plots showing the relationship between relative reduction in cell viability (x-axis) and ΔmiRNA expression (y-axis) across 21 primary glioblastoma cultures. Separate panels are shown for miR-21 (A), miR-26a (B), miR-34a (C), miR-181c (D), miR-181d (E), and miR-485-5p (F). A linear regression line with 95% confidence band is displayed onlyfor miR-26a, as this was the only miRNA exhibiting a significant association in the statistical analyses. Supplementary file1 (PDF 283 kb) [file 10014_2025_527_MOESM1_ESM.pdf]
